# Alisol A 24-acetate protects oxygen–glucose deprivation-induced brain microvascular endothelial cells against apoptosis through miR-92a-3p inhibition by targeting the *B-cell lymphoma-2* gene

**DOI:** 10.1080/13880209.2021.1912117

**Published:** 2021-04-27

**Authors:** Yangjie Zhou, Wei Wei, Julian Shen, Lu Lu, Taotao Lu, Hong Wang, Xiehua Xue

**Affiliations:** a The Affiliated Rehabilitation Hospital, Fujian University of Traditional Chinese Medicine, Fuzhou, China; b Rehabilitation Industry Institute, Fujian University of Traditional Chinese Medicine, Fuzhou, China

**Keywords:** MicroRNA-92a-3p, BMECs, Bcl-2

## Abstract

**Context:**

Alisol A 24-acetate has been used to treat vascular diseases. However, the underlying mechanisms still remain unclear.

**Objective:**

The present study evaluated the antiapoptotic effect of alisol A 24-acetate on brain microvascular endothelial cells (BMECs) and explored the underlying mechanisms.

**Materials and methods:**

BMECs were injured through oxygen -glucose deprivation (OGD) after alisol A 24-acetate treatment. Cell viability and half-maximal inhibitory concentration (IC_50_) were measured using CCK-8, whereas inflammatory factors and oxidative stress indicators were measured using enzyme linked immunosorbent assay. Cell invasion and wound healing assays were detected. Cell apoptosis was assessed using flow cytometry. B-cell lymphoma-2 (Bcl-2) and Bcl-2 associated X (Bax) expression were analyzed using Western blotting. Dual-luciferase assay was applied to detect target genes of miR-92a-3p.

**Result:**

Alisol A 24-acetate had an IC_50_ of 98.53 mg/L and inhibited cell viability at concentrations over 50mg/L. OGD induced apoptosis and promoted miR-92a-3p overexpression in BMECs. However, alisol A 24-acetate treatment suppressed inflammation, improved migration and invasion abilities, increased Bcl-2 expression, inhibited Bax expression, and repressed apoptosis and miR92a-3p overexpression in OGD-induced BMECs. MiR-92a-3p overexpression promoted cell apoptosis and suppressed Bcl-2 expression, whereas its inhibitor reversed the tendency. Alisol A 24-acetate treatment relieved the effects of miR-92a-3p overexpression. Dual-luciferase assay confirmed that miR-92a-3p negatively regulated the Bcl-2 expression.

**Conclusions:**

These findings suggest that alisol A 24-acetate exerts antiapoptotic effects on OGD-induced BMECs through miR-92a-3p inhibition by targeting the Bcl-2 gene, indicating its potential for BMECs protection and as a novel therapeutic agent for the treatment of cerebrovascular disease.

## Introduction

Ischaemic stroke, a major cerebrovascular disease defined as the sudden reduction in cerebral blood flow and insufficient oxygen supply to the brain tissues, is characterized by a series of cellular and molecular disturbances. The apoptosis of brain microvascular endothelial cells (BMECs) has been implicated in the occurrence and development of cerebral ischaemia (Zhang et al. 2005; Shin et al. 2016). It was reported that B-cell lymphoma-2 (Bcl-2) and Bcl-2 associated X (Bax) expression constituted the primary molecular mechanism underlying BMECs apoptosis after cerebral ischaemia (Li YN et al. 2014). Accordingly, apoptosis is inhibited with increased Bcl-2 expression and promoted with increased Bax expression (Strazielle and Ghersi-Egea 2013). Oxygen–glucose deprivation (OGD) induces cell apoptosis (Mo et al. 2016), during which Bax and Bax/Bcl-2 ratios are markedly upregulated (Zhao H et al. 2019). Bcl-2 and Bax proteins act as key apoptosis regulators (Czabotar et al. 2014), with their ratio being an important indicator of the process of apoptosis (Liu et al. 2017). Although several studies have suggested the importance of protective strategies for vascular endothelial cell apoptosis (Liu et al. 2016; Ross et al. 2016), no effective strategies and agents for treating vascular damage following ischaemic insult are available to date.

MicroRNAs (miRNAs) are small segments of noncoding sequences involved in the post-transcriptional regulation of gene expression (Bartel 2009). MiR-92a-3p, a member of the miR-17-92a cluster, is frequently dysregulated in various cancer types and plays a key role in cellular physiology (Li M et al. 2014). Moreover, miR-92a-3p is widely expressed in endothelial cells and is involved in endothelial cell apoptosis and vascular integrity (Rippe et al. 2012). Recently, several studies have revealed that miR-92a-3p was associated with cell apoptosis (Li 2019, Ling et al. 2020). However, the role miR-92a-3p plays in the apoptosis of cancer cells differs from that in ordinary cells. Endothelial cell injury can induce miR-92a-3p expression, indicating the possible involvement of miR-92a-3p in endothelial cell regulation and vascular function (Liu et al. 2019). As such, we speculated that miR-92a-3p is associated with endothelial cell apoptosis during OGD injury.

Alisol A 24-acetate, one of the main active components of *Alisma orientale* (Sam.) Juz. (Alismataceae), has been frequently used in the treatment of vascular diseases. In fact, our previous studies showed that *Alisma* and alisol A 24-acetate attenuated inflammation and oxidative stress (Xue et al. 2014, 2016), indicating their protective role against vascular diseases. To date, however, no study has yet investigated whether alisol A 24-acetate protects endothelial cells against apoptosis. Therefore, the present study sought to investigate the role of alisol A 24-acetate on OGD-induced mouse BMECs and to further explore potential mechanisms.

## Materials and methods

### Cell culture

Mouse BMECs (bEnd.3, Shanghai Jining Shiye Co. LTD) were cultured in high glucose Dulbecco’s modified Eagle’s medium (H-DMEM; Gibco, USA) supplemented with 10% foetal bovine serum (Gibco, USA) under 95% air and 5% CO_2_ at 37 °C. The culture medium was replaced once every 2–3 days.

### Cell viability

Alisol A 24-acetate (standard material, purity > 98%) was purchased from Shanghai Yuanye Bio-Technology Co. Ltd (Shanghai, China); dissolved in 50 μL dimethyl sulfoxide, with the final concentration of dimethyl sulfoxide in the medium being no more than 0.1%; and stored in the refrigerator at 4 °C for later use. Cells were incubated in H-DMEM at a density of 5 × 10^3^ cells per well in 96-well plates for 24 h. H-DMEM containing alisol A 24-acetate at various concentrations (0, 5, 10, 20, 40, 50, 80, 120, 140, and 160 mg/L) was then added. After 24 h of treatment, the cells were maintained in 10 μL/well CCK-8 solutions (BOSTER, Wuhan, China) for an additional 1 h and measured at 450 nm absorbance. Half maximal inhibitory concentration (IC_50_) was calculated using GraphPad Prism (v. 7.0).

### Oxygen–glucose deprivation

After reaching 80–85% confluence, cells were washed twice with phosphate buffer saline (PBS, Gibco, USA) and then randomly assigned to one of the following five groups: Control, OGD, OGD + 1 A, OGD + 10 A, and OGD + 20 A. The OGD group was established by culturing the cells with no glucose and serum-free basic salt solution. The plates were then placed in an anaerobic chamber at 37 °C with a controlled atmosphere of 5% CO_2_, 94% N_2_, and 1% O_2_ for 8 h. Thereafter, the cells were cultured with normal medium and placed back into the normal incubator for 24 h. Meanwhile, cells in the OGD + 1 A group, OGD + 10 A group, and OGD + 20 A group were established by pretreating the OGD group with 1, 10, and 20 mg/L alisol A 24-acetate for 24 h, followed by OGD stimulation, respectively.

### Superoxide dismutase and malondialdehyde assay

The cell culture supernatants were collected for measuring superoxide dismutase (SOD) and malondialdehyde (MDA) after OGD. The cell culture supernatants were centrifuged at 3000 rpm for 10 min at 4 °C and then collected for determination. Thereafter, the SOD levels were measured using the total SOD determination kit (WST-1 method) (Jiancheng Biotechnology, Nanjing, China). The MDA levels were tested using an MDA assay kit (TBA method) (Jiancheng Biotechnology, Nanjing, China) according to the manufacturer’s instructions.

### Determination of intracellular reactive oxygen species

Intracellular reactive oxygen species (ROS) were tested using an ROS assay kit (Beyotime Biotechnology). The intracellular ROS levels were measured by the fluorescent change resulting from oxidation of DCFH-DA, a fluorescent probe with membrane permeability. The cells were treated with DCFH-DA (10 μmol/L) at 37 °C for 20 min in the dark and washed three times with serum-free DMEM according to the manufacturer’s instructions. The fluorescent intensity of intracellular ROS was observed using a confocal laser microscope (UltraView VoX, PerkinElmer, USA) and measured using Image J.

### Analysis by enzyme-linked immunosorbent assay

Cell culture solutions were collected and centrifuged at 3000 rpm for 15 min, after which the supernatants were collected for analysis. Enzyme-linked immunosorbent assay (ELISA) for mouse tumour necrosis factor-alpha (TNF-α) and interleukin-1 beta (IL-1β) were performed according to the manufacturer’s instructions.

### Cell invasion assay

The cell invasion assay was performed using the Transwell chamber (BD BioCoat, Shanghai, China). Briefly, 300 µL of H-DMEM incubated at 37 °C was added to each Transwell chamber and left at room temperature for 20 min. After the intervention, cells were seeded into 24-well polycarbonate Transwell chambers with 8-μm pores at 1.5 × 10^4^ cells/well in 200 µL of serum-free medium, with the lower chamber being covered with 600 µL of the complete medium as a chemoattractant. After incubation for 32 h at 37 °C and 5% CO_2_, the noninvaded cells on the upper chamber were mechanically wiped off. The invaded cells were washed with PBS, immobilized in 4% paraformaldehyde for 5 min, dyed with 0.1% crystal violet for 20 min (Solarbio, Beijing, China), and quantified using six randomly selected fields of view under a Leica DFC295 microscope (Leica DFC295, Germany). Three independent experiments were conducted.

### Wound healing assay

Cells (1 × 10^5^/mL) were seeded into a 6-well plate in 2 mL of complete medium and treated as described earlier. A scratch was made using a 10 μL tip until the cells formed a confluent monolayer after OGD 8 h, after which the scratch was viewed and imaged under a Leica DFC295 microscope. After 24 h of culture, the closure of the scratch was viewed and imaged again. Three independent experiments were conducted. The percentage of the total cell-free zones was determined using six randomly selected fields of view to appraise cell migration capacity using Image J (National Institutes of Health).

### Flow cytometry

Annexin V-FITC apoptosis detection kit (KeyGen BioTECH, Jiangsu, China) was used to measure the percentage of apoptotic cells. Briefly, after being subjected to the necessary experimental treatments, cells were digested with trypsin, blown with the original medium, and collected through centrifugation. Cells were washed two times with PBS, resuspended with 500 μL of binding buffer (5 μL of Annexin V-FITC and 5 μL of propidium iodide), and fixed for 10 min away from light. A flow cytometer was used to determine the proportion of apoptotic cells within 1 h.

### Western blot analysis

BEnd.3 cells were digested with pancreatin, pipetted evenly, and then centrifuged. Thereafter, the supernatant was transferred to a 1.5 mL EP tube, lysed on ice with RIPA lysis buffer for 30 min, and fractionated thrice using ultrasound. The mixture was centrifuged at 12,000 rpm for 15 min, after which the supernatant was stored at 80 °C for subsequent experiments. The protein concentration was determined using the BCA assay, whereas protein expressions of Bcl-2 and Bax were determined using a Western blot assay. Briefly, the protein was mixed evenly with 6× loading buffer (5:1, v/v) and boiled for 10 min. Thereafter, 50 μg of total protein were separated using 10% polyacrylamide gel electrophoresis. The gel was transferred to a polyvinylidene fluoride membrane, blocked in 5% bovine serum albumin at room temperature for 2 h, and incubated with Bcl-2 (26 kDa, 1:1000, Proteintech), Bax (21 kDa, 1:5000, Proteintech), and mouse anti-mouse β-actin (42 kDa, 1:8000, Cell Signalling Technology) monoclonal antibodies at 4 °C overnight. The blot was washed thrice with TBST, incubated with horseradish peroxidase-conjugated goat anti-rabbit secondary antibody (1:5000, Beyotime Institute of Biotechnology; Shanghai, China) at room temperature for 1–2 h, washed thrice with TBST, and visualized using the ECL kit (Beyotime). Greyscale values of Western blot protein bands were estimated using Image-Lab v5.0 (Bio-Rad).

### RNA isolation and quantitative polymerase chain reaction

Total RNA was isolated from bEnd.3 cells using the RNAmisi microRNA rapid extraction kit (Aidlab Biotechnologies Co., LTD) and reverse transcribed into cDNA. The quantitative polymerase chain reaction was then conducted using the ABI 7500 Real-Time PCR System (Applied Biosystems, Foster City, CA, USA) with SYBR^®^ Premix Ex Taq™ II (Takara) according to the manufacturer’s instructions. miR-92a-3p expression was quantified using polymerase chain reaction assay under the following conditions: denaturation at 95 °C for 10 s, annealing at 60 °C for 30 s, 30 cycles of extension at 72 °C for 45 s, and, finally, extension at 72 °C for 7 min. U6 served as the internal reference. Primers were designed according to GenBank (Table 1). Cell miRNA levels were quantified using the 2^−ΔΔCt^ [ΔΔCt = (Ct miRNA1 − Ct U6) − (Ct miRNA2 − Ct U6)] relative quantification method using U6 as the internal control.

**Table 1.** The sequences of primers.

miRNA	Sequences
miR-92a-3p-F	5′-CCGCGTATTGCACTTGTCCC-3′
miR-92a-3p-R	5′-AGTGCAGGGTCCGAGGTATT-3′
U6-F	5′-CTCGCTTCGGCAGCACATATACT-3′
U6-R	5′-ACGCTTCACGAATTTGCGTGTC-3′

### Cell transfection

Mimics and inhibitors for MiR-92a-3p and NC were designed and synthesized by GenePharma (Shanghai, China). The sequences of the miR-92a-3p mimics and inhibitor, as well as the corresponding control (NC mimics) are detailed in Table 2. Cells were transfected using GP-transfect-Mate (GenePharma, Shanghai, China) according to the manufacturer’s protocol. Briefly, cells were seeded into 6-well cell culture plates at 1 × 10^5^ cells per well and grown to 60–70% confluency. Transfection complexes were prepared according to the manufacturer’s instructions and were added directly to the cells. The miR-92a-3p mimics (mi92a) and inhibitor (in92a), as well as the NC inhibitor (inNC) and mimics (miNC), were used at a concentration of 50 nM. Cells were then assigned randomly to seven groups: Control, OGD, miNC, inNC, mi92a, mi92a + 10 A, and in92a. The miNC, inNC, mi92a, and in92a groups were transfected, whereas the mi92a + 10A group was transfected respectively for 6 h, followed by treatment with 10 mg/L of alisol A 24-acetate for 24 h. After transfection, Bcl-2 and Bax expression in the OGD/R-induced bEnd.3 cells were measured.

**Table 2.** The sequences of miR-92a-3p mimics and inhibitor.

miRNA	Sequences
miR-92a-3p mimics	5′-UAUUGCACUUGUCCCGGCCU-3′
miR-92a-3pmimics NC	5′-UUCUCCGAACGUGUCACGUTT-3′
miR-92a-3p inhibitor	5′-CAGGCCGGGACAAGUGCAAUA-3′
miR-92a-3p inhibitor NC	5′-CAGUACUUUUGUGUAGUACAA-3′

### Validation of predictive target gene of miR-92a-3p

To verify Bcl-2 as targets of miR-92a-3p, wild-type and mutant-type 3′-untranslated regions (UTR) of Bcl-2 from mice were cloned into a Renilla luciferase control reporter vector (pRL-TK; Promega, USA). HEK-293 cells were co-transfected with miR-92a-3p mimics or mimic-negative controls and pRL-TK vectors using Lipofectamine^®^ 2000 (Invitrogen, USA). Firefly luciferase activity was detected using dual-luciferase assays (Genomeditech, Shanghai, China), which were normalized to a Renilla luciferase expression control.

### Statistical analysis

All experimental data from of three independent experiments were expressed as mean ± standard deviation. All statistical analyses were performed using SPSS version 21.0 (SPSS, Inc.; Chicago, IL, USA). One-way analysis of variance followed by Fisher’s protected least-significant difference test or Student’s *t*-test was used to compare two treatments, with a *p*-value of <0.05 indicating statistical significance.

## Results

### Cell viability after alisol A 24-acetate treatment

**Figure 1.**
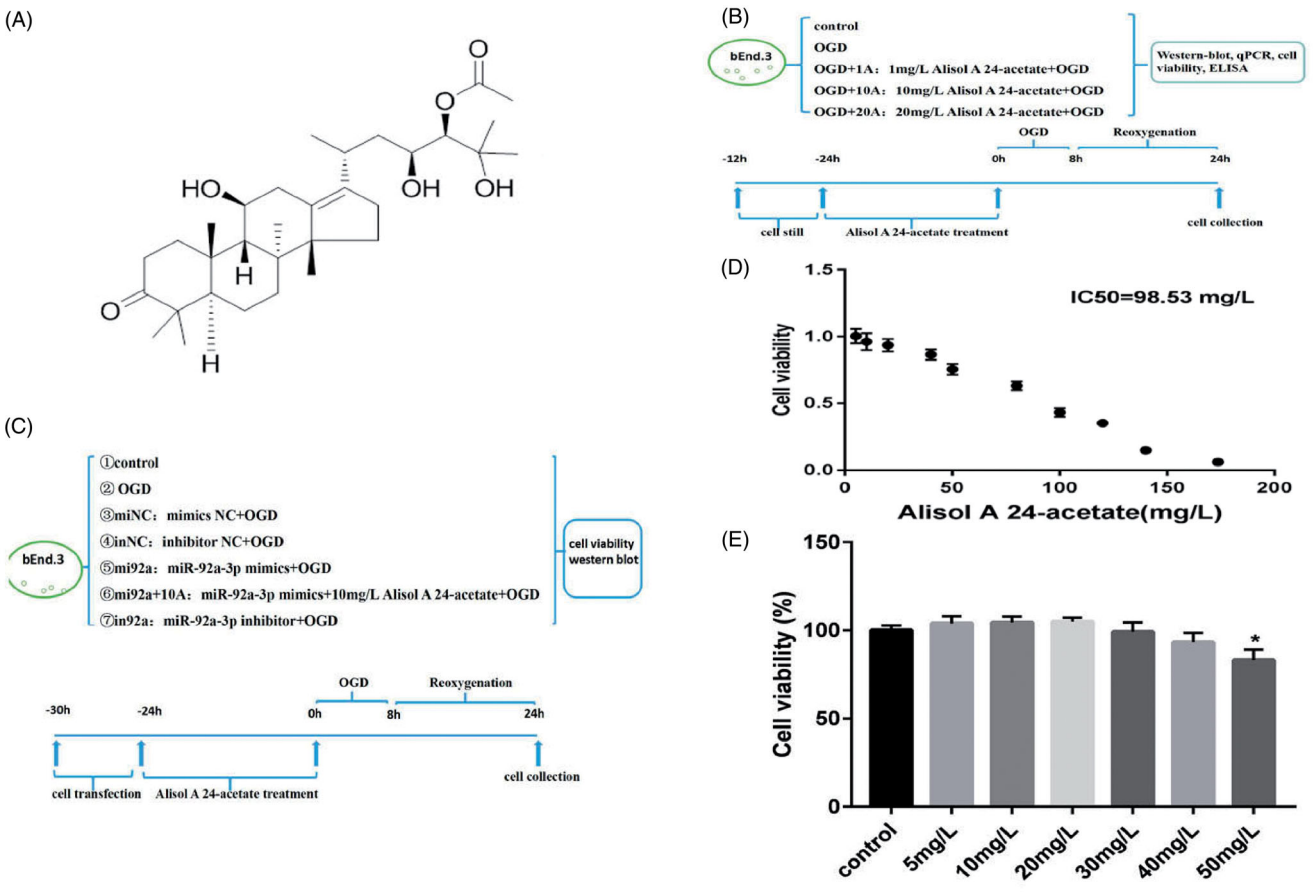
Cell viability after alisol A 24-acetate treatment. (A) Molecular structure of alisol A 24-acetate. (B, C) Experimental designs are displayed in panels (B) and (C). (D) Alisol A 24-acetate had an IC50 of 98.53 mg/L. (E) Cell viability after alisol A 24-acetate measured using the CCK-8 assay. Results are presented as means ± standard deviation (SD) (*n* = 6).

The molecular structure of alisol A 24-acetate and experimental designs are displayed in Figure 1(A–C). Cell viability was measured at concentrations ranging from 5 to 160 mg/L after alisol A 24-acetate treatment. Alisol A 24-acetate had an IC_50_ of 98.53 mg/L (Figure 1(D)) and inhibited cell viability at concentrations over 50 mg/L (*p* < 0.05, Figure 1(E)). Hence, 1, 10 and 20 mg/L of alisol A 24-acetate were selected in the present study.

### Alisol A 24-acetate repressed inflammation in OGD-induced bEnd.3 cells

**Figure 2.**
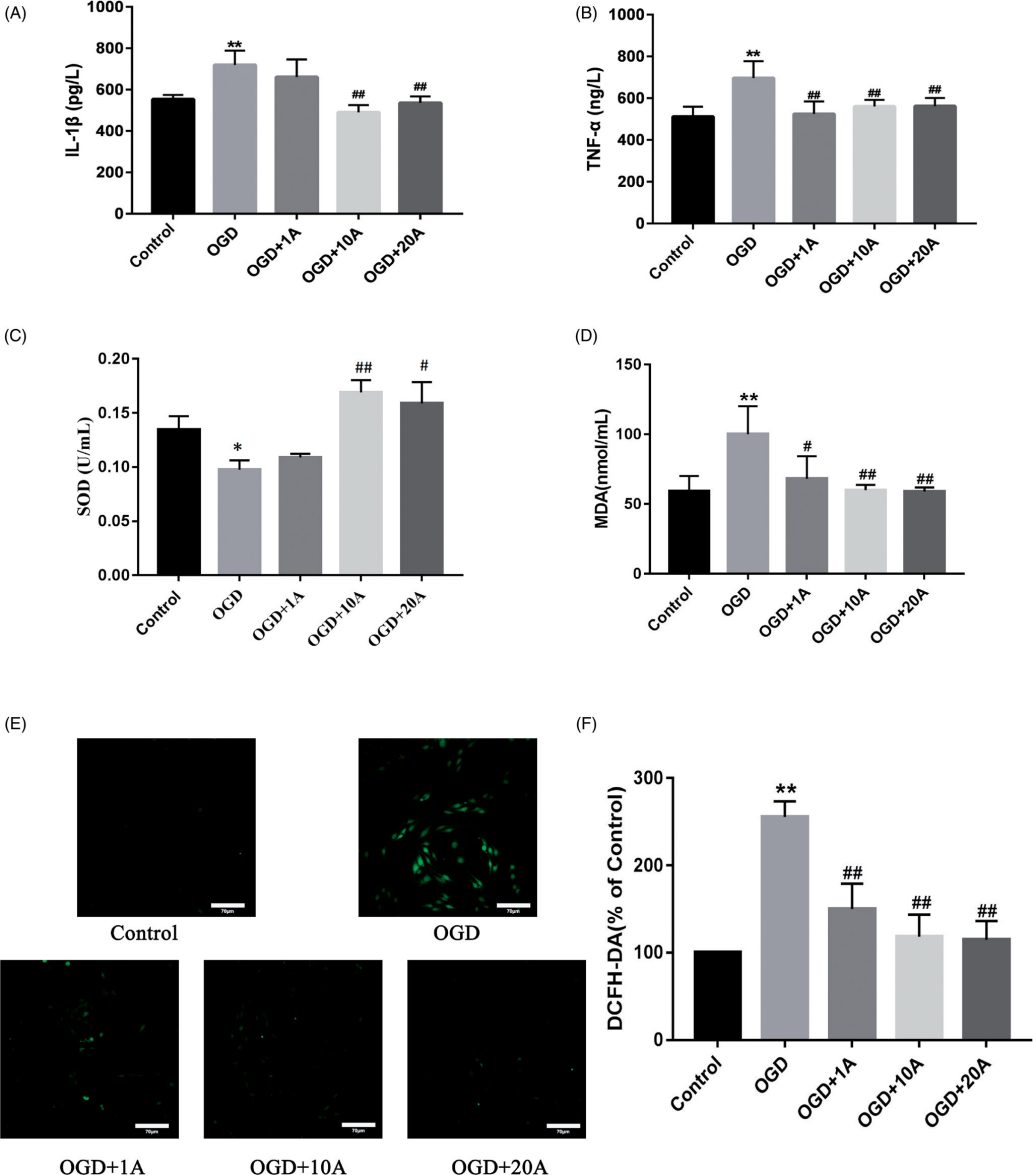
Effects of alisol A 24-acetate on inflammatory factors and oxidative stress in OGD-induced bEnd.3 cells. (A) Secreted IL-1β in cell culture supernatant was determined using ELISA. (B) Secreted TNF-α in cell culture supernatant was determined using ELISA. (C) Superoxide dismutase (SOD) activity in supernatant was detected. (D) Secreted malondialdehyde (MDA) in cell culture supernatant was determined. Results (A–D) are presented as means ± standard deviation (SD) (*n* = 6) vs. the control group (*p < 0.05, **p < 0.01), vs. the OGD group (^#^*p* < 0.05, ^##^*p* < 0.01). (E) The inhibitory effect of alisol A 24-acetate on OGD-induced ROS production. DCFH-DA, a fluorescent indicator, was used to visualise ROS, which was observed by confocal microscopy. Scale bar = 70 μm. (F) The quantity of DCFH-DA was measured by Image J software. Results are presented as means ± standard deviation (SD) (*n* = 3) vs. the control group (*p < 0.05, **p < 0.01), vs. the OGD group (^#^*p* < 0.05, ^##^*p* < 0.01).

ELISA was used to detect TNF-α and IL-1β levels in the cell culture supernatant. Our analysis showed that the OGD group had higher IL-1β and TNF-α levels than the control group (*p* < 0.01, Figure 2(A,B)). After treatment with 10 and 20 mg/L alisol A 24-acetate, IL-1β secretion decreased significantly (*p* < 0.01; Figure 2(A)). Moreover, alisol A 24-acetate (1, 10, and 20 mg/L) treatment repressed TNF-α expression in OGD-induced bEnd.3 cells (*p* < 0.01; Figure 2(B)).

### Alisol A 24-acetate affected oxidative stress in OGD-induced bEnd.3 cells

The SOD assay revealed that the OGD group had a significantly lower SOD activity than the control group (*p* < 0.01; Figure 2(C)). However, SOD activity was distinctly increased after alisol A 24-acetate treatment (10 and 20 mg/L) (*p* < 0.01 and *p* < 0.05, respectively; Figure 2(C)). MDA assay indicated that OGD induced the MDA expression (*p* < 0.01; Figure 2(D)) and alisol A 24-acetate treatment suppressed the expression of MDA dramatically (*p* < 0.01; Figure 2(D)).

### Alisol A 24-acetate inhibited intracellular ROS level in OGD-induced bEnd.3 cells

The ROS level was detected by DCFH-DA, a fluorescent reagent that acts as an ROS indicator. The results demonstrated that ROS level was significantly increased in the OGD group compared with the control group (*p* < 0.01; Figure 2(E,F)). Alisol A 24-acetate treatment significantly decreased the intracellular ROS level compared with the OGD group (*p* < 0.01; Figure 2(E,F)).

### Alisol A 24-acetate enhanced the invasion and migration capabilities of OGD-treated bEnd.3 cells

**Figure 3.**
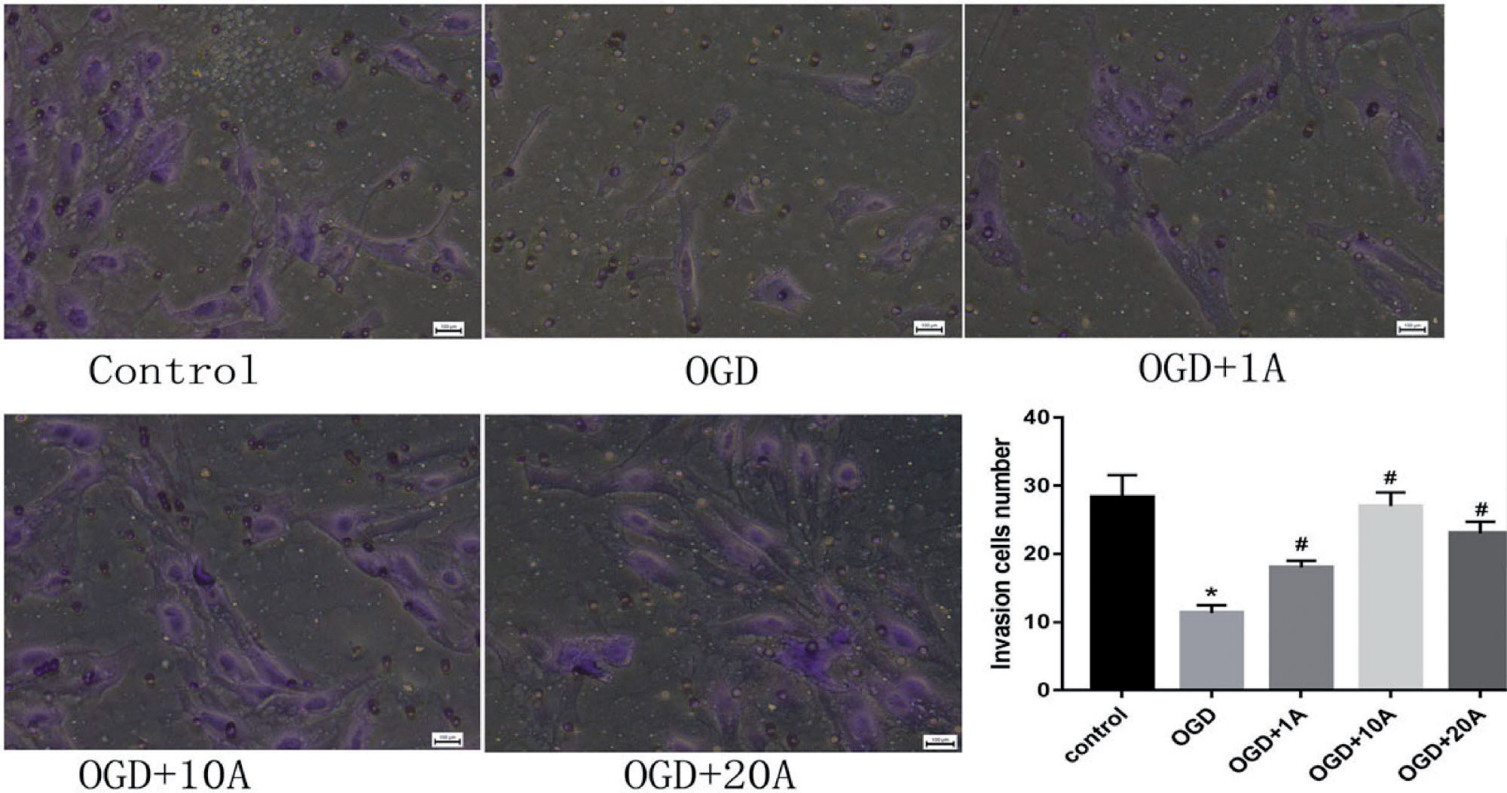
Effects of alisol A 24-acetate on invasion capabilities. Invasion capability was measured (*n* = 5). The ability of cells migration was measure (*n* = 5). vs. control group (*p < 0.05), vs. OGD group (^#^*p* < 0.05). Results are presented as means ± standard deviation (SD).

**Figure 4.**
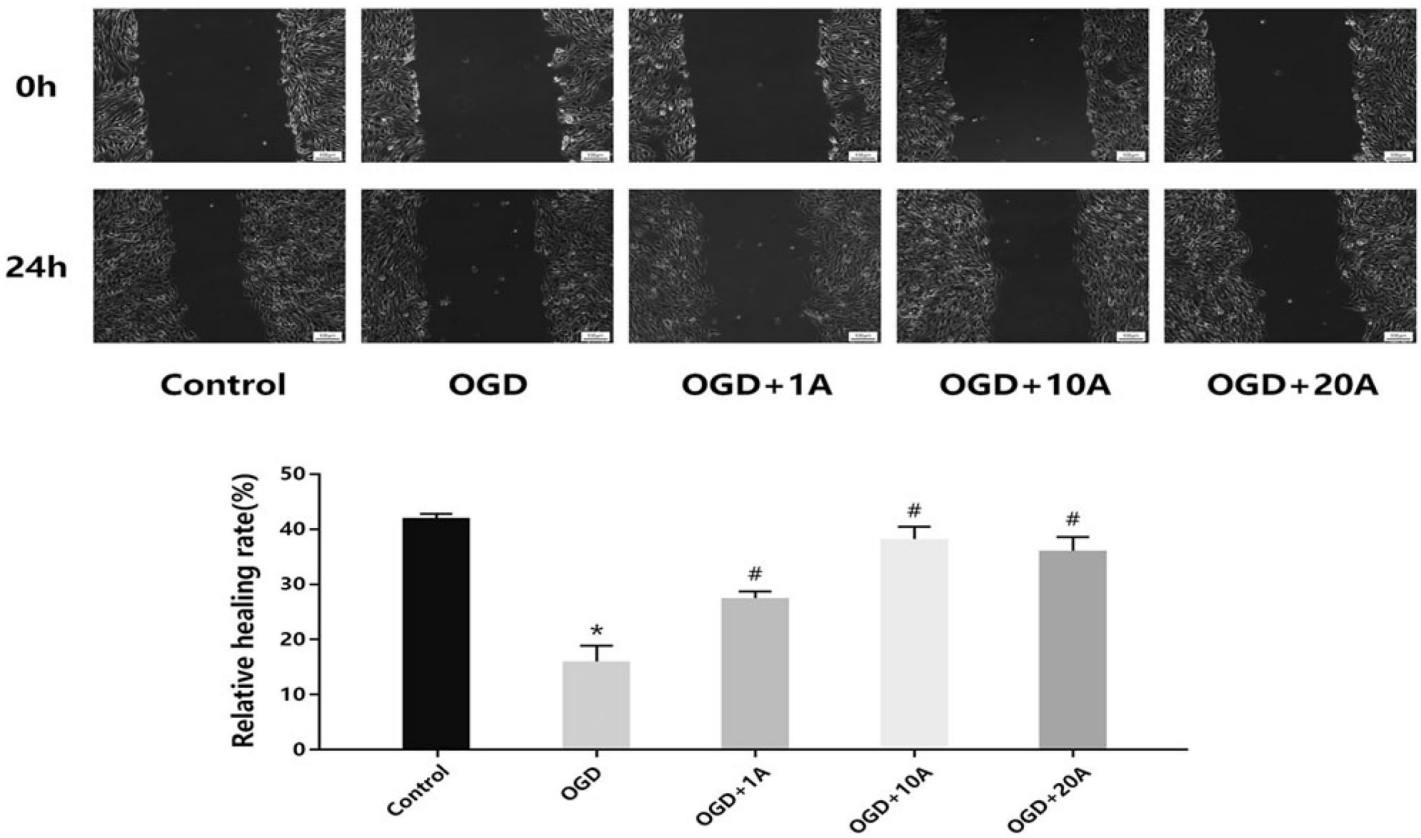
Effects of alisol A 24-acetate on migration capabilities. Cell migration ability was measured (*n* = 5). vs. the control group (*p < 0.05), vs. the OGD group (^#^*p* < 0.05). Results are presented as means ± standard deviation (SD).

The cell invasion assay showed that the OGD group had poorer invasion ability than the control group (*p* < 0.05; Figure 3), which subsequently increased following alisol A 24-acetate treatment (*p* < 0.05; Figure 3). The wound-healing assay found that alisol A 24-acetate improved the impaired migration capability in OGD-induced cells (*p* < 0.05; Figure 4).

### Alisol A 24-acetate inhibited apoptosis of OGD-induced bEnd.3 cells

**Figure 5.**
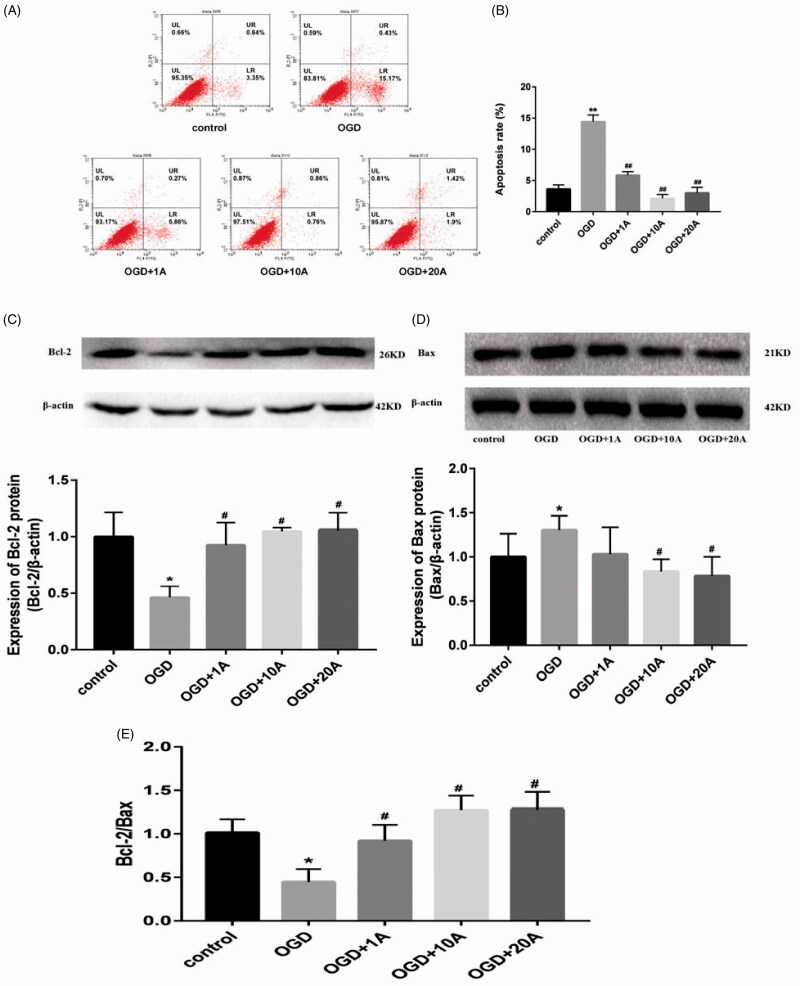
Effects of alisol A 24-acetate on apoptosis of OGD-induced bEnd.3 cells. (A) Apoptosis detected using flow cytometry. (B) Apoptotic ratio was calculated. (C) Western blot analysis to determine Bcl-2 expression. (D) Western blot analysis to determine Bax expression. (E) The Bcl-2/Bax ratio. The graphs represent the relative expression of these proteins over three independent experiments. vs. the control group (*p < 0.05, **p < 0.01), vs. the OGD group (^#^*p* < 0.05).

Flow cytometry results showed that alisol A 24-acetate (1, 10 and 20 mg/L) significantly suppressed the dramatically increased apoptotic ratio of OGD-induced cells (*p* < 0.01; Figures 5(A,B)). Western blot analysis revealed that OGD treatment repressed Bcl-2 expression, elevated Bax expression, and decreased the Bcl-2/Bax ratio (*p* < 0.05, Figure 5(C–E)), whereas alisol A 24-acetate treatment (1, 10 and 20 mg/L) increased Bcl-2 expression (*p* < 0.05; Figure 5(C)) at all dosages and inhibited Bax expression (*p* < 0.05; Figure 5(D)) at dosages of 10 and 20 mg/L. Alisol A 24-acetate treatment promoted a higher Bcl-2/Bax ratio than the OGD group (*p* < 0.05; Figure 5(E)), indicating that alisol 24-acetate exerts antiapoptotic effects in BMECs by regulating the Bcl-2/Bax expression.

### Alisol A 24-acetate affected miR-92a-3p expression of OGD-induced cells

**Figure 6.**
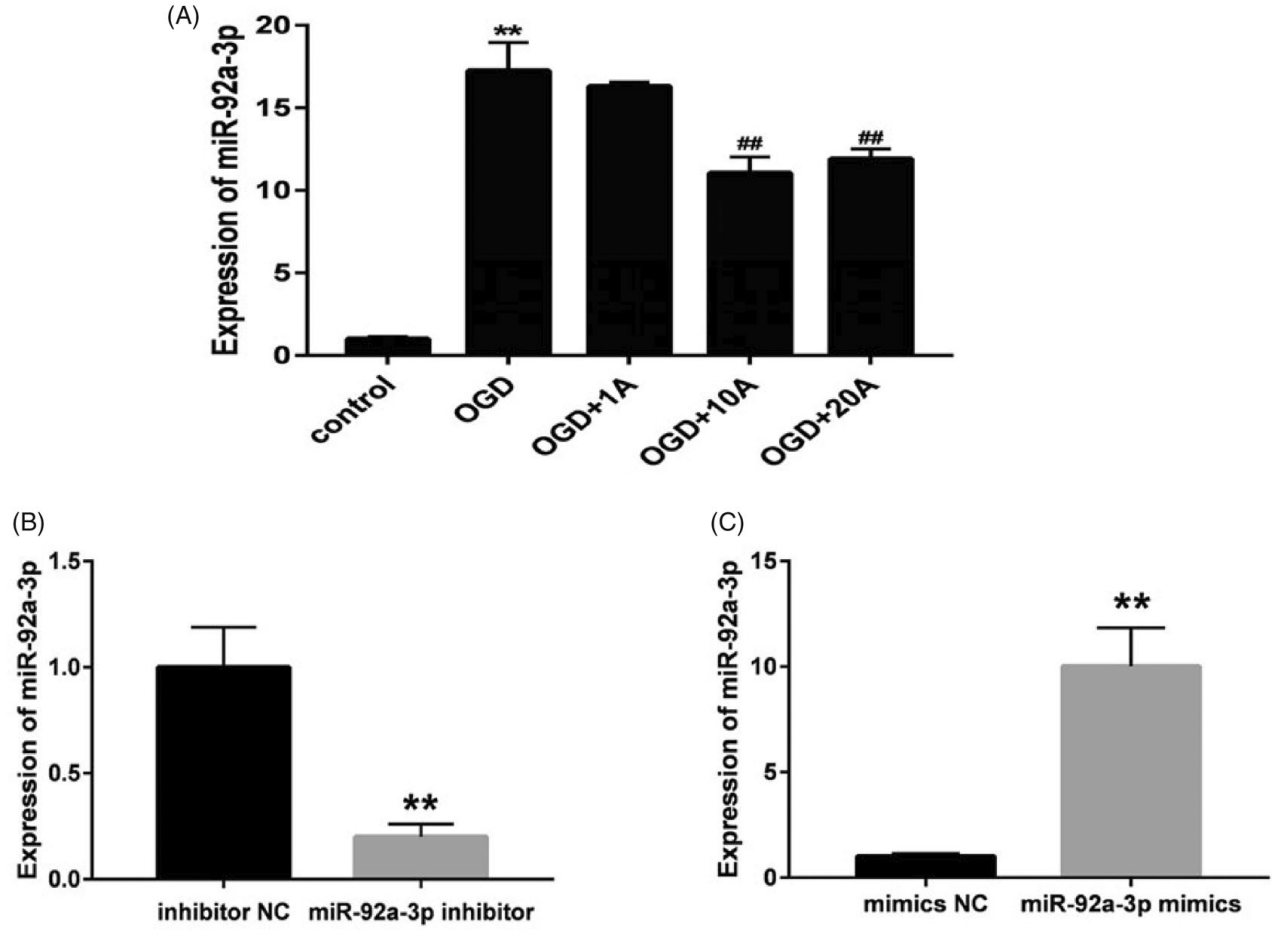
Effects of alisol A 24-acetate on miR-92a-3p expression of OGD-induced bEnd.3 cells. (A) MiR-92a-3p expression in cells detected using quantitative polymerase chain reaction. Results are presented as means ± standard deviation (SD) (*n* = 6). vs. the control group (**p < 0.01), vs. the oxygen–glucose deprivation group (^##^*p* < 0.01). (B) MiR-92a-3p expression after inhibitor and NC transfection (*n* = 6). (C) MiR-92a-3p expression after mimic and NC transfection (*n* = 6). vs. the NC group (**p < 0.01).

MiR-92a-3p expression was significantly increased after OGD injury (*p* < 0.05; Figure 6(A)). However, alisol A 24-acetate treatment inhibited miR-92a-3p overexpression after OGD injury (*p* < 0.05; Figure 6(A)).

### MiR-92a-3p transfection influenced the apoptosis of OGD-induced bEnd.3 cells

**Figure 7.**
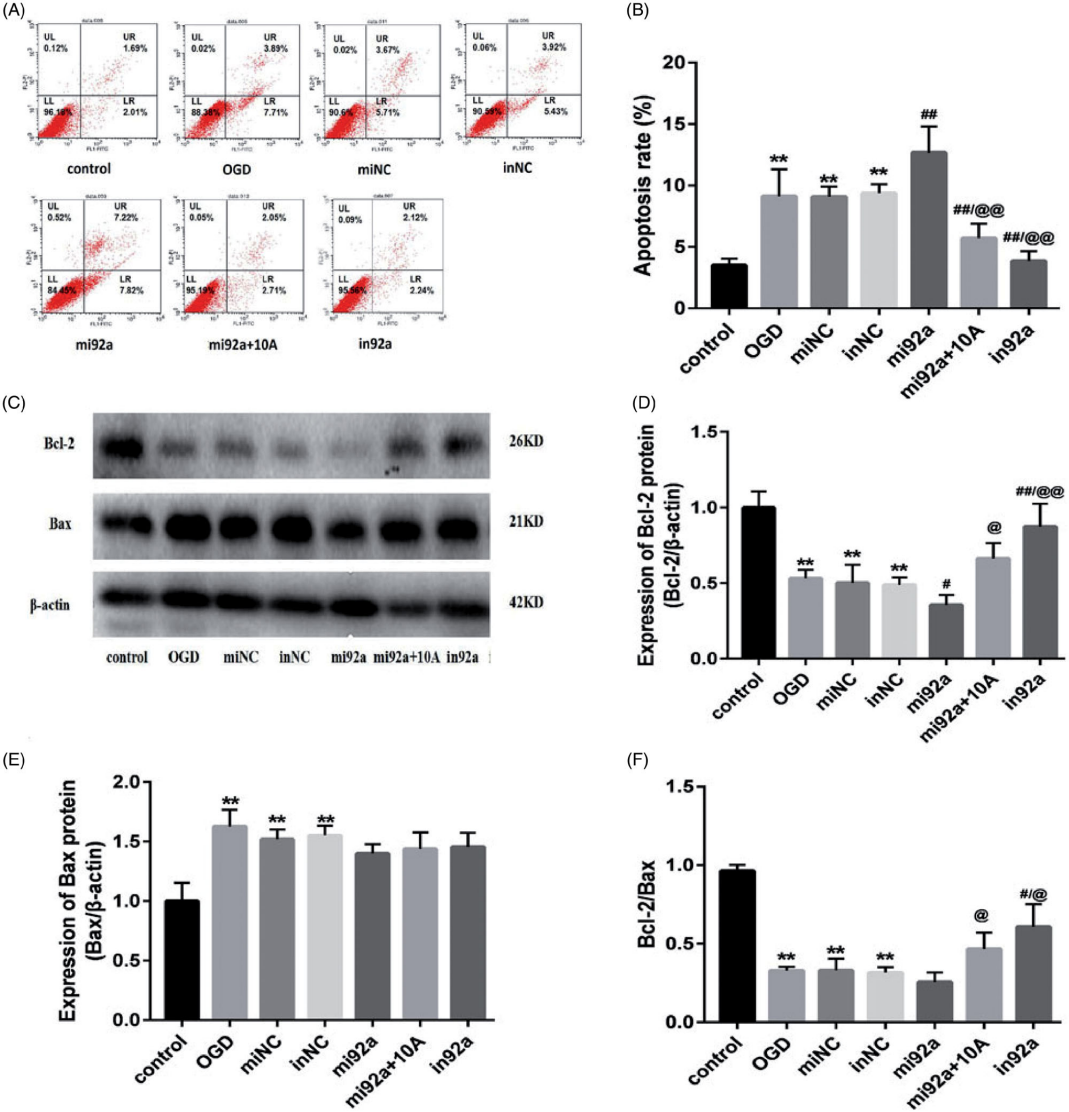
Effects of miR-92a-3p transfection on apoptosis of OGD-induced bEnd.3 cell. (A) Cell apoptosis detected using flow cytometry. (B) Apoptotic ratio was calculated. (C) Western blot analysis to determine of Bcl-2 and Bax expression, with β-actin serving as the loading control. (D) Bcl-2 protein expression. (E) Bax protein expression. (F) The of Bcl-2/Bax ratio. The graphs represent three independent experiments. Results are presented as means ± standard deviation (SD) (*n* = 3). OGD, miNC, and inNC vs. the control group (**p < 0.01); mi92a, mi92a + 10A, and in92a vs. the OGD group (^#^*p* < 0.05, ^##^*p* < 0.01); mi92a + 10A and in92a vs. mi92a group (^@^*p* < 0.05, ^@@^*p* < 0.01).

The miR-92a-3p mimic and inhibitor (50 nM) were transfected to explore the role of miR-92a-3p on OGD-induced cells. Accordingly, the miR-92a-3p mimic promoted higher miR-92a-3p expression, whereas the inhibitor promoted lower miR-92a-3p expression than the NC groups (*p* < 0.01; Figure 6(B,C)). Flow cytometric analysis of cell apoptosis after miR-92a-3p transfection (Figure 7(A)) found that the OGD and mi92a groups had a higher rate of cell apoptosis than the control group (*p* < 0.05; Figure 7(B)), indicating that miR-92a-3p overexpression aggravated apoptosis in the OGD-induced cells. The mi92a + 10 A (*p* < 0.05) and in92a groups (*p* < 0.01) had a lower percentage of apoptotic cells than the mi92a group (Figure 7(A,B)), suggesting that both miR-92a-3p inhibitor and alisol A 24-acetate (10 mg/L) exert antiapoptotic effects on OGD-injured cells by inhibiting miR-92a-3p expression.

Moreover, Western blot analysis and flow cytometry produced similar apoptosis-related protein (Bcl-2 and Bax) expression. Significant differences in Bcl-2 and Bax expression had been observed among the OGD, miNC and inNC groups and the control group (*p* < 0.01; Figure 7(C–F)). The miR-92a-3p mimic promoted higher downregulation of Bcl-2 expression in the mi92a group than in the OGD group (*p* < 0.05; Figure 7(D)), whereas the miR-92a-3p inhibitor promoted dramatically higher Bcl-2 expression in the mi92a group than in the OGD group (*p* < 0.01; Figure 7(D)). The mi92a + 10 A group had significantly higher Bcl-2 expression than the mi92a group, indicating that alisol A 24-acetate can elevate the Bcl-2 expression in OGD-induced cells by inhibiting miR-92a-3p expression. The miR-92a-3p mimic and inhibitor displayed unobvious inhibition of the Bax expression (Figure 7(E)), suggesting that miR-92a-3p may specifically regulate the Bcl-2 expression. The OGD, miNC and inNC groups had a lower Bcl-2/Bax ratio than the control group (*p* < 0.01; Figure 7(F)). The mi92a + 10 A and in 92a groups had gradually higher Bcl-2/Bax ratios than the mi92a and OGD groups (*p* < 0.05; Figure 7(F)), indicating that both alisol A 24-acetate and miR-92a-3p inhibitor showed antiapoptotic effects via directly or indirectly regulating Bcl-2 expression.

### MiR-92a-3p regulated Bcl-2 expression

**Figure 8.**
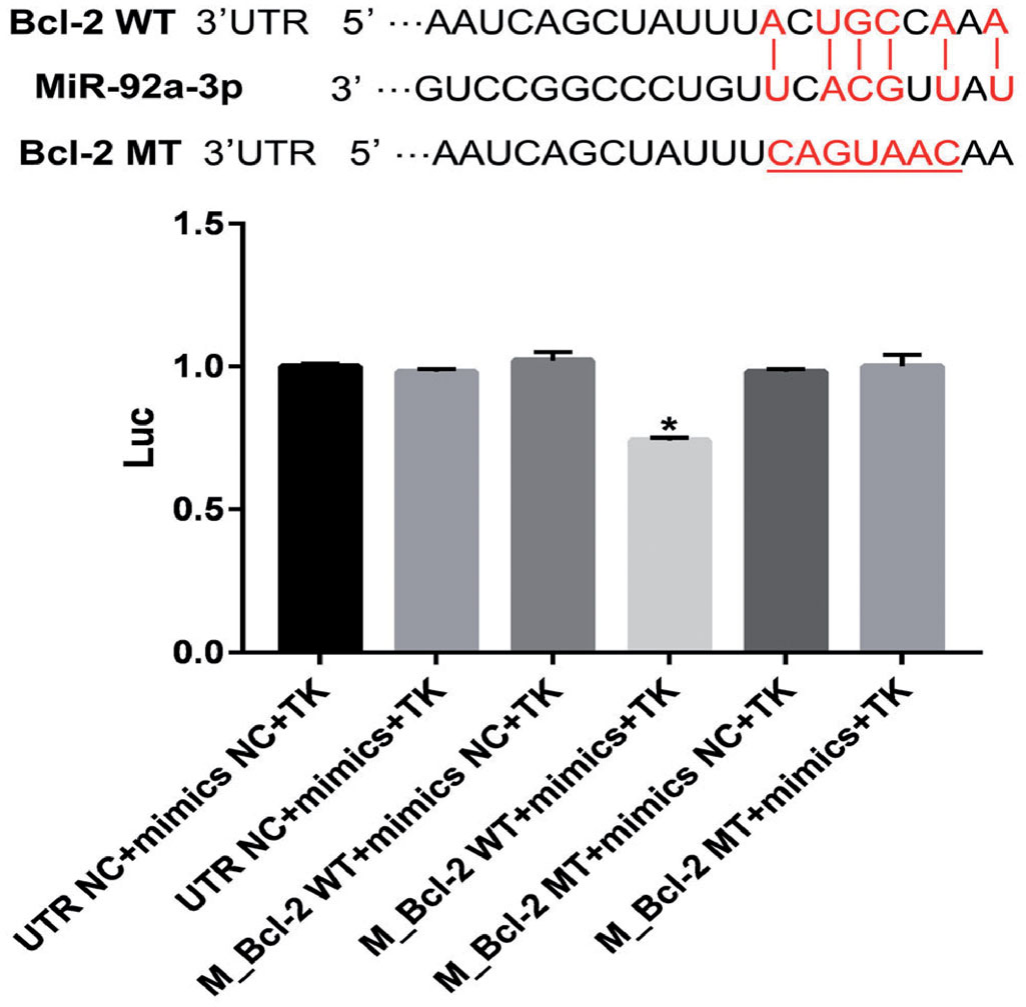
MiR-92a-3p directly targets *Bcl-2* gene. Luciferase activity of wild-type Bcl-2 3′-untranslated region (UTR) (WT) or mutant Bcl-2 3′-UTR (mut) with NC mimics or miR-92a-3p-transfected HEK-293 cells. The graphs represent three independent experiments. Results are presented as means ± standard deviation (SD) (*n* = 3) (*p < 0.05).

Bioinformatics analysis shows that miR-92a-3p binds to the 3′-UTR noncoding sequences of the *Bcl-2* gene. To explore the role of miR-92a-3p on the regulation of Bcl-2, HEK-293 cells were co-transfected with a Renilla luciferase reporter vector containing the wild-type Bcl-2 3′-UTR (WT) and mutant Bcl-2 3′-UTR, UTR NC, and mimic NC. Accordingly, our results showed that Bcl-2 3′-UTR (WT)-transfected cells had higher luciferase activity than the control groups (*p* < 0.05; Figure 8). However, miR-92a-3p promoted no difference in luciferase activity in cells containing mutant Bcl-2 3′-UTR (mut) and NC controls (Figure 8). Thus, the provided data demonstrate that the binding sites of miR-92a-3p in the Bcl-2 3′-UTR sequence are critical for miR-92a-3p function. Altogether, miR-92a-3p binds directly to the Bcl-2 3′-UTR sequence, suggesting that *Bcl-2* is the target gene of miR-92a-3p.

## Discussion

Ischaemic stroke has remained one of the main causes of death among adults. Impairment in BMEC integrity following a stroke is one of the important pathophysiological mechanisms of cerebral ischaemia. Inflammatory factors and apoptosis play an important role in BMECs destruction. Therefore, it is important to focus on the protection of BMECs to improve defective symptoms after cerebral ischaemia.

Alisol A 24-acetate, one of the main active components of *Alisma*, is a triterpenoid isolated from the tubers of *A. orientale*. Interestingly, reports have demonstrated that alisol 24-acetate inhibited the expression of ROS and inflammatory factors through the AMPK/mTOR pathway (Wu et al. 2018). Moreover, alisol A 24-acetate regulates inflammatory factors (Xue et al. 2016). As such, we speculated that alisol A 24-acetate exerts a protective effect on OGD-induced BMECs and that it can be used as an optional approach for protecting BMECs. Accordingly, our results showed that alisol A 24-acetate exerted protective effects by mitigating apoptosis, as well as by promoting migration and invasion capabilities in OGD-induced BMECs.

Cerebral ischaemia induces oxidative stress and inflammatory responses, which promote cell apoptosis (Raha and Robinson 2001; Liu et al. 2015; Zhao et al. 2019; Zhao et al. 2019). Oxidative stress has been proved to be a consequence of disequilibrium between the generation of ROS and oxidation resistance (Newsholme et al. 2016). A previous study reported that ROS was regarded as the most important indicator of oxidative stress, which led to induce endothelial cell apoptosis and vascular damage (Incalza et al. 2018). MDA is the end-product of lipid peroxidation triggered by excessive ROS, mainly considered as a late marker of cellular injury (Salmanoglu et al. 2016; Verma et al. 2019). SOD, an endogenous scavenger of oxygen-free radical, has been shown to remove excessive ROS production and maintain a balance between oxidation and antioxidation (Lin and Beal 2006). Production of ROS and MDA was widely used to reflect the level of oxidative stress; conversely, SOD, which acts as an antioxidant, is essential to decrease the overproduction of ROS and MDA to prevent vascular injury and cell death (Cui et al. 2020). OGD induces inflammatory responses and oxidative stress by increasing the levels of TNF-a, IL-1β, IL-6, ROS and MDA and decreasing SOD activity (Zhao et al. 2019). Moreover, TNF-α and IL-1β can induce endothelial cell apoptosis and inflammation (Iadecola and Anrather 2011). The present study found that OGD suppressed SOD activity and induced TNF-α, IL-1β, ROS and MDA expression; however, alisol A 24-acetate treatment promoted an obvious increase in SOD activity and repression of TNF-α, IL-1β, ROS and MDA expression in the OGD-induced cells.

Apoptosis, a form of programmed cell death that plays a key role in homeostasis by eliminating injured cells, is the major form of BMECs death following brain ischaemic injury (Mattson et al. 2001). Cerebral ischaemia induces endothelial cell inflammation and accelerates cell apoptosis (Desideri et al. 2003; Miao et al. 2017). Bcl-2 is the antiapoptosis gene that serves as a negative regulator of cell death (Dietrich 1997; Kvansakul et al. 2017), whereas Bax is one of the apoptosis-promoting genes (Cao et al. 2017). Both Bcl-2 and Bax are the primary genes involved in BMECs apoptosis (Bedirli et al. 2012). Previous studies have revealed that activation of antiapoptotic Bcl-2 protein or inactivation of pro-apoptotic Bax protein can disturb the process of vascular endothelial cell apoptosis under ischaemic conditions (Dietrich 1997; Bedirli et al. 2012; Cao et al. 2017; Kvansakul et al. 2017; Miao et al. 2017). Our data showed that although OGD increased the number of apoptotic cells and decreased Bcl-2 expression and the Bcl-2/Bax ratio, alisol A 24-acetate treatment increased Bcl-2 expression, repressed Bax expression, and attenuated the apoptosis in the OGD-induced cells. The present findings provide evidence that alisol A 24-acetate displayed notable protective effects in OGD-induced BMECs by inhibiting apoptosis.

The present study revealed that while OGD induced inflammation/oxidative stress and apoptosis, which impaired the migration and invasion capabilities of BMECs, treatment with alisol A 24-acetate attenuated apoptosis, as well as increased cell migration and invasion capabilities in OGD-induced cells.

Cumulative evidence has demonstrated that miRNAs regulate the pathophysiology of endothelial cells (Bonauer et al. 2009; Bonauer and Dimmeler 2009; Daniel et al. 2014). MiR-92a-3p, which is highly expressed in endothelial cells (Li et al. 2019), has been linked to endothelial cell injury (He et al. 2017) and becomes overexpressed in brain tissues and plasma of rats during ischaemia and hypoxia (Gusar et al. 2017). The present study found that while OGD induced miR-92a-3p overexpression in BMECs, alisol A 24-acetate treatment inhibited its expression in OGD-induced cells. To further verify the function of miR-92a-3p in apoptosis, miR-92a-3p mimics and inhibitors were transfected to BMECs. Accordingly, our results showed that miR-92a-3p overexpression induced cell apoptosis but was reversed by miR-92a-3p inhibition and alisol A 24-acetate treatment. This finding indicated that miR-92a-3p was associated with cell apoptosis and that the miR-92a-3p inhibitor, as well as alisol A 24-acetate, exerted an antiapoptotic effect in BMECs during OGD injury. Taken together, our data showed that alisol A 24-acetate can repress the apoptosis of OGD-induced cells by inhibiting miR-92a-3p expression.

It has been demonstrated that miR-92a-3p is associated with apoptosis and that the inhibition of miR-92a-3p suppresses pancreatic cell apoptosis (Ling et al. 2020), a finding similar to that presented herein. The present study showed that miR-92a-3p mimics suppressed Bcl-2 expression, whereas the miR-92a-3p inhibitor increased it. However, both had no effect on the Bax expression, indicating that miR-92a-3p regulates cell apoptosis by directly or indirectly inhibiting the Bcl-2 expression. Bioinformatics analysis showed that miR-92a-3p binds to the 3′-UTR noncoding sequence of the *Bcl-2* gene according to prediction using base-pairing rules. The present study cloned the 3′-UTR binding sites of the Bcl-2 mRNA fragment and the mutant 3′-UTR fragment containing the putative miR-92a-3p binding sites upstream of the luciferase coding sequence and performed co-transfection of the luciferase reporter. Subsequent results showed that miR-92a-3p regulated the Bcl-2 expression by binding to the 3′-UTR sequences of the *Bcl-2* gene, further suggesting that alisol A 24-acetate exerts antiapoptotic effects on OGD-induced BMECs through miR-92a-3p inhibition by targeting the *Bcl-2* gene.

## Conclusions

Alisol A 24-acetate suppresses apoptosis of OGD-induced BMECs through miR-92a-3p inhibition by targeting the *Bcl-2* gene. The present study provides new insights into the underlying mechanisms through which alisol A 24-acetate protects BMECs.
